# A tale of two lexica: Investigating computational pressures on word representation with neural networks

**DOI:** 10.3389/frai.2023.1062230

**Published:** 2023-03-27

**Authors:** Enes Avcu, Michael Hwang, Kevin Scott Brown, David W. Gow

**Affiliations:** ^1^Department of Neurology, Massachusetts General Hospital, Harvard Medical School, Boston, MA, United States; ^2^Harvard College, Boston, MA, United States; ^3^Department of Pharmaceutical Sciences and School of Chemical, Biological, and Environmental Engineering, Oregon State University, Corvallis, OR, United States; ^4^Athinoula A. Martinos Center for Biomedical Imaging Massachusetts General Hospital, Charlestown, MA, United States; ^5^Department of Psychology, Salem State University, Salem, MA, United States; ^6^Harvard-MIT Division of Health Sciences and Technology, Boston, MA, United States

**Keywords:** mental lexicon, word representation, neural networks, functional segregation, dorsal and ventral streams, deep learning

## Abstract

**Introduction:**

The notion of a single localized store of word representations has become increasingly less plausible as evidence has accumulated for the widely distributed neural representation of wordform grounded in motor, perceptual, and conceptual processes. Here, we attempt to combine machine learning methods and neurobiological frameworks to propose a computational model of brain systems potentially responsible for wordform representation. We tested the hypothesis that the functional specialization of word representation in the brain is driven partly by computational optimization. This hypothesis directly addresses the unique problem of mapping sound and articulation vs. mapping sound and meaning.

**Results:**

We found that artificial neural networks trained on the mapping between sound and articulation performed poorly in recognizing the mapping between sound and meaning and vice versa. Moreover, a network trained on both tasks simultaneously could not discover the features required for efficient mapping between sound and higher-level cognitive states compared to the other two models. Furthermore, these networks developed internal representations reflecting specialized task-optimized functions without explicit training.

**Discussion:**

Together, these findings demonstrate that different task-directed representations lead to more focused responses and better performance of a machine or algorithm and, hypothetically, the brain. Thus, we imply that the functional specialization of word representation mirrors a computational optimization strategy given the nature of the tasks that the human brain faces.

## 1. Introduction

In 1865, Paul Broca declared the left-third frontal convolution of the brain to be the “center of articulate speech”. In the years that have followed, cognitive neuroscientists have embraced an increasingly granular and differentiated view of localized cognitive function. Multiple brain regions have been associated with particular cognitive functions thanks to advances in imaging techniques and the functional decomposition of cognitive processes. Examples include the visual word form area (VWFA), which responds to orthographic forms (Dehaene et al., 2002; Dehaene and Cohen, 2011), the fusiform face area (FFA), which is specialized for facial recognition (Kanwisher et al., 1997), and the extrastriate body area (EBA), which responds selectively to images of the human body (Downing et al., 2001). Even more recently, a neural population has been identified in the human auditory cortex that shows selective sensitivity to singing (Norman-Haignere et al., 2022). These findings invite the question of why such narrow sensitivities arise in the first place. In this paper, we investigate one such localized function, the functional specialization of wordform representation, by utilizing a neuro-inspired machine learning approach. Our premise is that task-directed representations are essential to perform a task (Bengio et al., 2013). We propose that input data characteristics will force the machine/algorithm, and hypothetically, the brain, to discover the representations required for feature detection. Thus, we examine whether the functional specialization of wordform representation is driven partly by computational constraints that are inherent in the mapping between spoken words and their evoked cognitive states.

**Figure 1:**
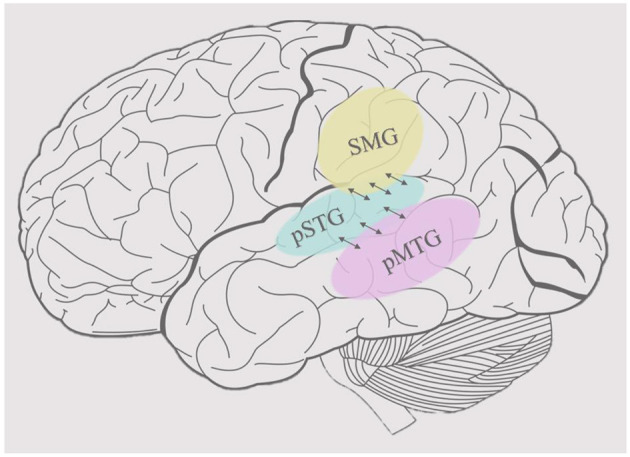
Cortical organization of wordform representation. The posterior superior temporal gyrus (pSTG), shown in light blue, is the main area of acoustic-phonetic processing of natural speech. The supramarginal gyrus (SMG), shown in yellow, mediates the mapping between acoustic-phonetic representations and articulation, whereas the posterior middle temporal gyrus, shown in pink, mediates the mapping between acoustic-phonetic representations and meaning.

Wordform representation, the stored representation of the sound patterns of words, has been associated with the bilateral posterior middle temporal gyrus (pMTG) and adjacent posterior temporal regions (the ventral stream network) in addition to the supramarginal gyrus (SMG) and adjacent inferior parietal areas (the dorsal stream network) (Hickok and Poeppel, 2007; Gow, 2012) (see Figure 1). Words provide a useful level of representation for organizing the processing in both streams, but it is not clear why two distinct wordform networks evolved. The development of speech and language probably relied on neural systems that were already present in other primates' brains which are organized dually, similar to those in the visual cortex (Rauschecker, 1998). Thus, the division between dorsal and ventral processing streams appears to predate the evolution of language in both visual and auditory processing (Rauschecker and Scott, 2009; Sheth and Young, 2016). This suggests that dorsal and ventral processing divergences constrain the modern functional organization of spoken language processing. In addition to these potential anatomical constraints, we hypothesize that computational constraints of input data shaped the development of parallel wordform networks that rely on different featural representations of words to mediate different mappings between sound and higher-order linguistic representations.

Words play a crucial computational role in language by mediating the mapping between sound patterns and cognitive states. Elman (2004) and Gow (2012) describe the computational role of words as being functionally equivalent to hidden nodes in a neural network, providing representations that optimize the mapping between signal structure and the cognitive traits they evoke. In a sense, words are provisional representations in service of understanding meaning and articulation. The dorsal and ventral processing architecture impose two very different sets of demands on wordform representation. While complex, the dorsal stream mapping between sound and articulation is relatively systematic, temporally contiguous, and primarily dependent on identifying segmental units of the phoneme or syllable. In contrast, the ventral stream mapping between sound and meaning is partially systematic, largely arbitrary, and primarily dependent on identifying morphological units that frequently span multiple segments. From the perspective of the dorsal stream, the words *cats* and *cast* might be viewed as single-phoneme insertion neighbors of *cat*. From the perspective of the ventral system, *cat* and *cats* are closely related, while *cat* and *cast* are quite different. Given these differences, we hypothesize that these mappings would depend on different featural representations of wordform. In this respect, the existence of two parallel networks raises the following fundamental questions: How do computational constraints of input data contribute to the emergence of these parallel streams? Do the computational demands of dorsal vs. ventral stream processing require different featural representations of wordform to accomplish efficient mapping?

Neuro-inspired machine learning techniques such as artificial neural networks (ANNs) provide a useful tool for exploring these questions (LeCun et al., 2015). These techniques are opening up unprecedented ways of thinking about how the brain works, specifically within the domains of perception, vision, and cognition (Yamins and DiCarlo, 2016; Flesch et al., 2018; Geirhos et al., 2018; Rajalingham et al., 2018; Zhou and Firestone, 2019; Golan et al., 2020). One such example is reinforcement learning, a type of machine learning inspired by the brain's reward system that uses positive and negative feedback to guide the learning process (Sutton and Barto, 2018). Recent reviews also suggest that deep neural networks (DNNs), which are composed of many hierarchically organized layers of ANNs, have the potential to completely remodel the way we think about neural computations (see Kriegeskorte, 2015; Marblestone et al., 2016; Bowers, 2017; Lake et al., 2017; Cichy and Kaiser, 2019; Saxe et al., 2021). Algorithms, such as DNNs, use a hierarchical combination of non-linear functions to transform raw input into more complex features, allowing for the identification of new patterns and improved performance on tasks such as image recognition and natural language processing (Bengio et al., 2013).

In the present study, we used long short-term memory (LSTM) architectures (Hochreiter and Schmidhuber, 1997) to test the hypothesis that the complex but systematic mapping between sound and articulation in the dorsal stream places different demands on feature sets than the more arbitrary mapping between sound and meaning. Any system (either machine or brain) learning to map sound to articulatory information vs. sound to meaning will intrinsically develop a different feature set because the characteristics of these mappings are different. Namely, the dorsal mapping depends on identifying segmental units (phonemes, syllables), whereas ventral mapping depends on morphological units. We created three LSTM networks to test this hypothesis and trained them independently on the same set of auditory word tokens. A dorsal network was trained to map words onto vectors representing whole-word articulatory properties so that the level of linguistic description that it is capturing would be phonological. In contrast, a ventral network was trained to map words onto vectors reflecting broad distributional semantic properties to capture semantic content. We also created a “fused” model that was trained on both tasks simultaneously to provide a direct comparison of the computational efficiency of parallel vs. single-stream wordform mapping. All words had unique sparse output representations. After training, we extracted patterns of network activation from the hidden layer of each network and tested how well the features extracted from one model supported the classification of input based on articulatory vs. semantic properties. We predict that: (i) Features from a dorsal LSTM model trained on dorsal mappings should have an advantage for articulatory categorization but not semantic categorization, (ii) Features from a ventral LSTM model trained on ventral mappings should have an advantage for semantic categorization but not articulatory categorization, and (iii) Features from a fused LSTM model trained on both dorsal and ventral mappings should not have an advantage for categorization related to articulatory or semantic categorization, compared to the specialized models. It should be noted that this study does not use neural data to test the spatial localization of wordform representation in the brain. Instead, we use computational modeling to investigate the computational constraints that could have caused the brain to develop two parallel word processing systems.

## 2. Cortical organization of wordform representation

In one of the founding works of the neurobiology of language, Wernicke (1874/1969) inferred the existence of a *wortshatz* (“treasury of words”) in the posterior superior temporal lobe from an association between localized damage in aphasia and impaired auditory speech comprehension. Wernicke's concept of the wortshatz is similar to Pustejovsky's (1998) notion of a sense enumeration model, in which words are bundles of stored information describing meaning, syntactic function, and phonological form. Within this framework, word recognition or lexical comprehension deficits reflect the loss of this enumerated knowledge. Rather than focusing on the role of the word as a mediating representation, work on the neural bases of lexical knowledge focused mainly on the distributed localization of lexically indexed semantic knowledge grounded in motor, perceptual, and conceptual processes [see a review by Patterson et al. (2007)] and possible dissociations between input and output lexica (Jacquemot et al., 2007).

The search for a lexical interface area began with Hickok and Poeppel (2004), who hypothesized the existence of a single sound-meaning interface broadly localized to the left temporal, parietal, and occipital cortex junction that maps semantic representations to acoustic-phonetic representations. Later versions of the dual-stream model postulated the bilateral posterior middle temporal gyrus (pMTG) as the lexical interface region (Hickok and Poeppel, 2007) and cortices adjacent to the posterior inferior temporal sulcus (pITS) as components of the ventral stream. Hickok and Poeppel associated this lexical interface region in the ventral stream with a lemma level of representation. Bilateral pMTG has also been shown to play a role in both regular and irregular morphological processes (Joanisse and Seidenberg, 2005; Tyler et al., 2005; Yokoyama et al., 2006). These findings contributed to conceptualizing this region as a house of wordform representation with morphological properties rather than a store of semantic knowledge. Hickok and Poeppel propose that representations stored in bilateral pMTG connect semantic representations and syntactic processes stored in a broad, bilateral distributed network with the acoustic-phonetic representations localized in bilateral posterior superior temporal gyrus (pSTG). With hypothesized bidirectional information flow within the ventral processing stream, this interface area plays a role in both the production of spoken words to communicate meaning and the interpretation of words spoken in context.

Evidence that the pMTG plays a role in ventral stream lexical processing comes from transcortical sensory aphasia (TSA). TSA generally occurs following posterior and/or inferior temporal lobe damage and involves impaired auditory comprehension with preserved syntactic and phonological abilities (Kertesz et al., 1982). Furthermore, electrical stimulation studies of speech/language abilities (Lüders et al., 1991), imaging studies of semantic processing (Binder et al., 2000), studies finding word comprehension deficits in Wernicke's aphasia (Baker et al., 1981), and neuropsychological studies that focus on word-level semantic deficits (Hart and Gordon, 1990) support the ventral lexicon's aforementioned roles in mapping between sound and meaning [see Hickok and Poeppel (2004) for a detailed review].

Gow (2012)'s dual lexicon model extends the dual-stream model of language processing and synthesizes evidence from aphasia, behavioral, and neural results to identify a second wordform area. In the dorsal processing stream, the left SMG (the inferior portion of Brodmann's area 40 delineated by the intraparietal sulcus, primary intermediate sulcus, the postcentral sulcus, and the Sylvian fissure) and the adjacent parietal operculum mediate the mapping between sound and word-level articulatory representation. This dorsal lexicon is hypothesized to play roles in speech production and perception, articulatory working memory rehearsal, and storage of articulatory organized wordform representations. Behavioral evidence for this claim includes the separable effects of semantic and wordform priming (Gaskell and Marslen-Wilson, 2002; Misiurski et al., 2005; Norris et al., 2006), lexical biases in non-word repetition errors (Vitevitch and Luce, 2005), the influence of word-level properties including lexical frequency (Vitevitch and Luce, 1998, 1999, 2005) and phonological neighborhood properties on articulation (Munson and Solomon, 2004; Scarborough, 2004; Wright, 2004; Munson, 2007), and syllabic encoding effects on speech production (Cholin et al., 2006).

Evidence from functional MRI studies investigating BOLD sensitivity to whole word properties similarly shows that lexical frequency and neighborhood (Shallice et al., 2000; Goldrick and Rapp, 2007; Knobel et al., 2008; Romani et al., 2011), competitor environment (Prabhakaran et al., 2006; Righi et al., 2010; Peramunage et al., 2011), lexical suppression and enhancement (Graves et al., 2007; Buchsbaum and D'Esposito, 2009), and word learning (Cornelissen et al., 2004; Mechelli et al., 2004; Green et al., 2007; Lee et al., 2007; Richardson et al., 2010) are modulated by the inferior parietal lobe, particularly the SMG, in addition to the area that Hickok and Poeppel (2007) hypothesized to be the lexical interface. Within this framework, the lexically preserving phonological paraphasias (Yamadori and Ikumura, 1975) seen in reproduction conduction aphasia (Shallice and Warrington, 1970; Vallar and Baddeley, 1984) following inferior parietal damage may be attributed to the degradation of dorsal lexical representations, just as semantic paraphasia seen in transcortical sensory aphasia following damage to posterior middle temporal regions may be attributed to the degradation of ventral lexical representations (Wernicke, 1874/1969; Goldstein, 1948; Coslett et al., 1987).

To the extent that Gow's dual lexicon model explains a wide range of empirical results, it also raises a fundamental question: Why do humans need stored representations of wordforms in two parallel streams? We hypothesize that this seemingly unparsimonious redundancy stems in part from the pre-language evolution of separate dorsal and ventral auditory processing and the general usefulness of words as units of meaning and articulation. However, we also suspect that computational constraints imposed by the structure of spoken language and the divergent goals of the dorsal and ventral speech streams also contribute to this organization. In summary, the primary function of the wordform representations stored in the dorsal and ventral streams is to act as an interface between low-level representations of sound and higher-level representations of different aspects of linguistic knowledge, such as meaning and articulation.

## 3. Computational rationale of the model

While the term deep learning might be new, the use of neural networks to test the theories of neural computation related to language processing dates to the 1980s' parallel distributed processing models [see reviews by McClelland and Rogers (2003)]. Early models were used to explore the role of single vs. multi-stream mapping related to problems including the reading of words with regular vs. irregular orthography and the formation of regular vs. irregular forms of the English past tense [see McClelland and Patterson (2002), Pinker and Ullman (2002), and Westermann and Ruh (2012) for an overview]. These models played a significant role in shaping these debates but were limited in several respects. Chief among them was their reliance on training sets that did not reflect the distributional properties of real-world input and empirically unsupported assumptions about the form of input representations. Significantly, both limitations had the potential to bias the computational adequacy of the learning mechanisms that were the primary intended focuses of the work.

Deep learning models have made recent substantial progress in the perception and production of language, which is an ability generally attributed to humans (Chomsky, 2006; Turing, 2009; Dehaene et al., 2018). Transformer based models (Rothman, 2021), in particular, can comprehend, condense, translate, and generate text that closely aligns with the given prompt with a high degree of precision (Vaswani et al., 2017; Devlin et al., 2019; Brown et al., 2020; Floridi and Chiriatti, 2020). Moreover, deep learning models have been shown to process linguistic units (syllables, words, sentences) to the extent that is similar to the human brain (Lake and Murphy, 2021; Caucheteux and King, 2022; Hale et al., 2022). Such findings are only possible with the extraction of task-directed representations. Representation learning models acquire valuable representations, such as those that can be easily understood, possess hidden characteristics, or can be applied in transfer learning (Bengio et al., 2013).

Several recent studies have used convolutional neural networks (CNNs) originally developed for image processing (Le Cun et al., 1989; Gu et al., 2018) to explore task-optimized feature spaces for the classification of naturalistic inputs and their implications for functional specificity in cortical processing [(Kell et al., 2018; Kell and McDermott, 2019; Dobs et al., 2022; Kanwisher et al., 2023a); see Kanwisher et al. (2023b) for a review]. Others have used LSTMs to explore the emergent representation of temporally structured inputs and have found essential convergences with human neural representations (Magnuson et al., 2020).

Kell et al. (2018) investigated whether deep CNNs trained on speech (identification of words presented in noise) and music (identification of musical genre presented in noise) tasks show human-like error patterns or predicted patterns of neural response to the same stimuli. CNNs are a class of deep learning models inspired by early neural visual processes and are typically applied to image classification, object detection, text detection and recognition, action recognition, and scene labeling (Le Cun et al., 1989; Gu et al., 2018). By effectively spatializing the temporal structure of auditory input by converting audio input into psychophysically biased cochleagrams, Kell and colleagues were able to take advantage of CNNs' strengths as image classifiers. Their models classified both types of input with high accuracy, showing patterns of confusion that correlated strongly with human performance. More importantly, their models predicted voxel-level BOLD activation in human fMRI data, with a significant correlation between activity at sequential layers of the model and analogous regions in the auditory neural processing stream. These findings reflect hierarchical auditory processing and differentiation between higher-level processing of speech and non-speech stimuli. This work is an important step toward capturing a more accurate view of the computational problems posed by auditory word recognition. By training their models on natural speech, Kell and colleagues were able to capture critical aspects of the inherent variability of the speech signal, including variability related to speaker and rate. These results and subsequent related works have demonstrated the potential of using deep learning methods to explore the role of task-optimal processing to generate and test hypotheses about neural representations and the functional organization of the brain (Kell et al., 2018; Kell and McDermott, 2019; Dobs et al., 2022; Kanwisher et al., 2023a).

One area where this work falls short is in the handling of the temporal structure of auditory speech. The transience of the speech signal and the rate of spoken communication place significant constraints on speech processing (Marslen-Wilson and Tyler, 1980) that are not captured by CNN modeling. Magnuson et al. (2020) address these limitations by using a two-layer LSTM network (Hochreiter and Schmidhuber, 1997) trained on the mapping of multiple-talker synthetic speech to pseudo-semantic outputs. Like human listeners, LSTMs receive input moment-by-moment and make continuous processing commitments based on incomplete information. LSTMs are a type of recurrent neural network (RNN) that solve the problems of vanishing or exploding gradients (Bengio et al., 1994) using an architecture with three internal gates and a storage output gate. This architecture helps LSTMs find and exploit long-range temporal dependencies and makes them natural models for temporally structured tasks like speech recognition (Graves et al., 2004, 2013a,b). Magnuson et al. (2020)'s shallow model demonstrated high accuracy on the problem of recognizing individual words based on a speech from multiple talkers. Furthermore, despite training their model with arbitrarily distributed vectors to distinguish individual words, analysis of hidden node activity revealed that their model discovered phonetic features that corresponded closely to features represented in the superior temporal gyrus. Elman (1990, 2004) reports similar results related to the hidden unit sensitivities of simple recurrent networks (sRNNs). Elman trained a sRNN on a succession of sentences where words were fed to the network one by one, and the network's task was to predict the next word. Although the network was not explicitly trained to identify the grammatical class of individual words, analyses of hidden node feature space revealed clustering based on the grammatical and semantic properties of individual words. Elman's assumption was that the network used distributional information to induce categories such as noun, verb, or animacy.

## 4. Materials and methods

### 4.1. Training data

**Figure 2:**
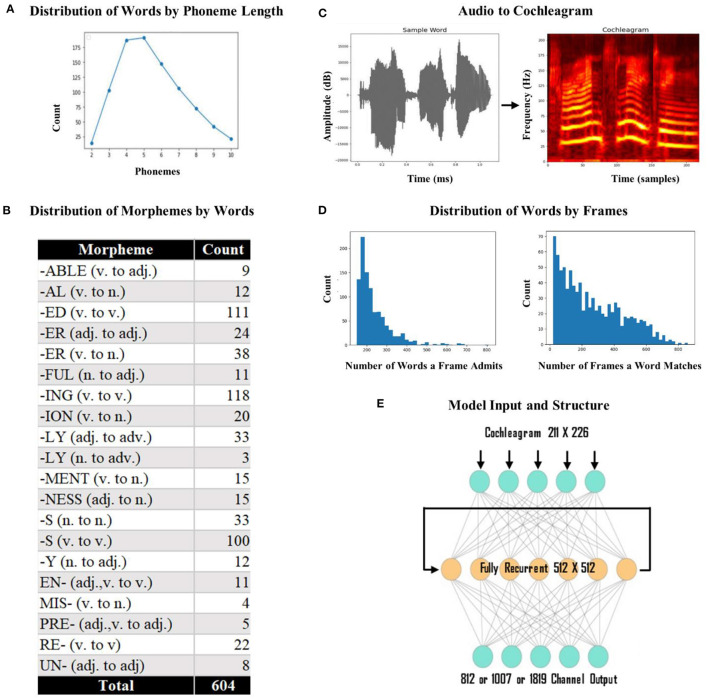
Model input and structure. **(A)** Distribution of words by phoneme length. We had a total of 883 words with lengths varying from 2 to 10 phonemes. Most of the words had four or five phonemes. **(B)** Distribution of morphemes by word count. The most frequently used affix was the suffix –“ing” (v. to v.) with 118 words and the least frequently used affix was “-ly” (n. to adv.) with 3 words. **(C)** Conversion of sample audio to a cochleagram. The *x-axis* represents the time (1,130 ms) and time samples (226), and the *y-axis* represents the amplitude (dB) and frequency (211 Hz). **(D)** Frequency distribution of the number of words a template admits and the number of templates a word matches. The number of words a template admitted was at least 160, and the number of templates a word matched was at least 20. **(E)** The model architecture. The model was a standard recurrent LSTM network with 512 fully recurrent hidden nodes. The output layer of the model was a dense layer with the sigmoid function, either with 812 (dorsal) or 1,007 (ventral) output vectors and 1,819 vectors for the fused network.

We used individual words as the input to the network rather than words in sentences because we wanted to isolate discourse level effects. We began with a set of 260 phonetically diverse monomorphemic English words. Since our aim is to investigate whether ventral stream mapping is dependent on identifying morphological units, we introduced morphology into our lexicon. We used 20 of the most commonly used English affixes (15 suffixes and 5 prefixes) to generate inflected words derived from the monomorphemic words (i.e., derived *fathers* from *father* using the plural inflectional morpheme –*s*). At the end of this process, we generated 1000 words: 260 monomorphemic words, 690 words in root form with one affix, and 50 words in root form with two affixes. To limit the variance that would be caused by the difference between short words and long words, we applied a form length constraint between 2 to 10 phonemes and ended up with 883 words (mean form length 5.5) as the final lexicon (see Figure 2A for distribution of words by phoneme length). These 883 words included 252 roots, 604 words in root form with one affix (see Figure 2B for the distribution of affixes by word), and 27 words in root form with two affixes. This final lexicon includes each of the 39 phonemes found in standard American English. We used the Apple text-to-speech program Say to generate pronunciations (audio) for all the words in our lexicon. This program provides a library of potential voices and relies on a unit selection and concatenation strategy to create naturalistic speech. This strategy has the advantage of capturing differences between speakers that might not be fully captured by parameter-based synthesis. We used 10 different speakers (five females and five males) to ensure a diverse set of tokens for each word (each word has 10 tokens, making a total of 8,830 total training items). The mean utterance duration was 684 ms (range: 335–1,130 ms).

We used cochleagrams of each sound file as the input to the network (Kell et al., 2018; Feather et al., 2019). A cochleagram is a spectrotemporal representation of an auditory signal designed to capture cochlear frequency decomposition (i.e., it has overlapping spectral filters whose width increases with center frequency). We used cochleagrams to provide the input to the model in a format similar to the way the brain gets the sound input (cochleagrams are more physiologically realistic than spectrograms). To create cochleagrams, we first trimmed any silence surrounding each word (with a cutoff of −20 dB) from the audio files. Each sound clip was passed through a bank of 203 bandpass filters that were zero-phase with center frequencies ranging from 50 Hz to 8,000 Hz. To perfectly tile the spectrum so that the summed squared response across all frequencies was flat, four low-pass and four high-pass filters were included [see Kell et al. (2018) for a detailed review], which led to a total of 211 filters. After determining the longest (in time) cochleagram in the set, we padded each input with empty values, so all cochleagrams were of equal length; we used a masking layer in the network that ignores any padded values (i.e., clamps the activity during the pads). This process resulted in a cochleagram representation of 226 x 211 (time x frequency) cells. See Figure 2C for a schematic representation of audio to cochleagram conversion. Cochleagrams were created in Python, using the *numpy* and *scipy* libraries (Oliphant, 2007; Harris et al., 2020), with signal trimming *via librosa* (McFee et al., 2015).

### 4.2. Training tasks

We created three separate LSTM models and trained them independently on the same training data (8,830 tokens for 883 words). A “dorsal” network was trained to differentiate between words using vectors representing articulatory properties, and a “ventral” network was trained to distinguish words based on semantic properties. In addition, a “fused” network was trained to distinguish words based on combined articulatory and semantic properties.

We chose the dorsal task to draw attention to whole-word articulatory properties without explicitly requiring sublexical segmentation into phonemes or syllables. To do this, we created target vectors using a variation on PatPho (Li and MacWhinney, 2002). PatPho is a slot-based system that represents words as an initial consonant cluster (CCC) followed by VVCCC blocks for each syllable. All vectors have as many blocks as are necessary to encode the longest word. Each C and V slot in the word is filled by a phonetic feature vector or a similarly sized vector of zeros. The longest word in our lexicon has five syllables; therefore, the length of the longest word in the lexicon in terms of the number of slots would be 28 (three slots for the initial consonant cluster and 25 slots for each syllable). We used 29-dimensional binary feature vectors for our encodings. In sum, for the lexicon in our study, every word vector was of length 812 (29 X 2*8*). For example, the word *cable* (/kabL/), in a lexicon in which no words had more than one syllable, would be k00a0bL0, where “0” means the appropriately sized vector of zeros. Words shorter than the maximum syllable length have trailing zeros to fill all remaining slots. In this way, the model doesn't get any temporal cues as to which parts of the vector are active at which time. With such a task, the dorsal network would use whole-word articulatory properties for efficient sound-to-articulation mapping.

The ventral network was trained to differentiate between words represented by sparse vectors derived from corpus-based lexical co-occurrence statistics. Such vectors are widely used as a surrogate for meaning representation in distributional semantics (Mandera et al., 2017; Lenci, 2018) because semantically similar words tend to occur in similar linguistic contexts. Representations formed from distributional semantic models (DSMs) *via* various transformations (such as reweighting) have been shown to be better than raw count-based models (e.g., Bag-Of-Words) (Baroni and Lenci, 2010). However, this unsupervised and independent vector transformation process often produces output vectors that are extremely large and very sparse. Word embedding models [e.g., Word2Vec (Mikolov et al., 2013)], another type of DSM based on learned (either supervised or unsupervised) representations of meaning, resolve these limitations by learning a distributed representation for words and produce low-dimensional vectors with dense features. Baroni et al. (2014) compared the performance of word embedding models with count-vector-based distributional semantic models and concluded that the former performed better on most tasks. Here, we have not used supervised word embedding models because we wanted our feature vectors to be dense but, at the same time, more interpretable. Thus, we used a mixed approach that created static word embeddings *via* a count-based approach that tries to avoid exceedingly large vectors by providing a more interpretable mapping between the sound input and its distributional properties.

We constructed word templates using the Corpus of Contemporary American English (COCA) n-grams data (Davies, 2010). We used 3-gram sequences to represent word meaning instead of a full sentence because we wanted to limit co-occurrence statistics to three words (a sentence can be longer or shorter than three words). COCA includes 16.3 million within sentence 3-gram sequences. We treated each 3-gram phrase as a template to constrain the place where a word can occur. Each template took the form of a 3-gram slot, and the target word could fill any of the three slots. For example, “__ as a” is a template that admits words like “act” (i.e., “act as a” is a valid 3-gram in the corpus). Each word in our lexicon can occur in the initial (i.e., “act as a”), middle (i.e., “to act for”), and final (i.e., “way to act”) positions. Each word in our lexicon had a predefined grammatical class (noun, verb, adjective, adverb) assignment based on its most frequent grammatical class. We did not use templates that have only variables. To control for sense ambiguity, we restricted templates based on the grammatical class of the target word within the template. If a word has more than one grammatical class associated with it (i.e., *act* can be a noun and a verb), we limited our templates to the most frequent grammatical class. For example, only templates in which the word *act* served as a verb were included in the set of 3-grams that defined the sparse vector for *act*. Moreover, since a word gets its meaning from the context (template) in which it occurs, we control for polysemy and homonymy.

Our 883 words occurred with more than forty thousand templates. To limit the number of templates and encourage generalization across words, we wanted each word to occur in at least 20 templates, and we only used templates that admitted at least 160 words (these limits were meaningful in our lexicon, but for a different lexicon, these limits might change). This resulted in a total of 1,007 templates; see Figure 2D for the distribution of words by the template. These limits on templates ensure low-dimensional vectors. For each word, we generated a sparse target vector with n of 1,007 selected elements set to 1 (all other elements 0), where n is the number of times a specific word pairs with a specific template (the same target vector was used for each of the tokens of a word). In the end, a vector space of length 1,007 represents the meaning of a word in n different contexts. This way (using the most frequent (1,007) global contexts based on word trigrams), we ensured denser (compared to classic count-based models) and more interpretable (compared to word embedding models) vectors. With this task, the ventral network would use broad-level co-occurrence statistics of words for efficient sound-to-meaning mapping.

The fused network was trained on combined dorsal and ventral sparse vectors creating sparse vectors consisting of 1,819 elements (812 replicating dorsal encoding and 1,007 replicating ventral encoding).

### 4.3. State-of-the-art comparison and network architecture

While most recent approaches to modeling task-optimized representations have utilized CNNs (Kell et al., 2018; Kell and McDermott, 2019; Dobs et al., 2022; Kanwisher et al., 2023a,b), we used LSTMs to model functional specialization of wordform representation in the brain. This preference is mainly because of the temporal structure of auditory speech data. LSTM is a type of recurrent neural network that is designed to remember past inputs and outputs for a longer period of time. This allows LSTMs to better handle sequential data, such as time series data or natural language. On the other hand, CNN is a type of neural network that is designed to process images and other grid-like data. If the task at hand is to model the visual processing system of the brain [i.e., the functional specialization of face perception in the brain as in Dobs et al. (2022), Kanwisher et al. (2023a,b)], then CNNs would be a better fit for the task. However, LSTMs would be a better model when the task is to capture long-term dependencies in language data. Additionally, LSTMs have a mechanism called “gating” which allows them to selectively choose which information to keep and which to discard in a sequential manner, which is similar to the way the brain processes and filters information. Finally, it has been shown that shallow LSTM models demonstrated high accuracy on the task of recognizing individual words based on a speech from multiple talkers and discovered phonetic features that corresponded closely to features represented in the STG of the brain (Magnuson et al., 2020).

There are many architectural and hyperparameter choices when building neural networks. It has been shown that the optimization of these hyperparameters can substantially affect the training performance of the network (Pinto et al., 2009; Yamins et al., 2014; Zoph et al., 2017). Hyperparameter tuning may include changing the number or types of layers, the choice of optimization algorithm, the use of dropout or other forms of regularization, tuning the learning rate/schedule, adjusting the batch size, and several other factors. Choosing a model architecture that supports accurate performance on a similar task is also important (Razavian et al., 2014). To this end, the number of recurrent layers, as well as the number of nodes in each layer, and numerous other parameters were determined through extensive hyperparameter tuning (see Supplementary Table S1 and Supplementary Figure S1 for details). We present results for the single best hyperparameter setting.

The final model consisted of 3 layers: (i) a masking layer, (ii) a hidden layer with 512 LSTM nodes, and (iii) a dense layer with random sparse vector outputs (812 for the dorsal, 1,007 for the ventral, and 1,819 for the fused network). See Figure 2E for the final structure of the network. The 226 x 211 cochleagrams were first passed to a masking layer. For a model, the input data must be a single tensor of shape batch_size x time x frequency. After padding, all the cochleagrams had a uniform length. The masking layer ensured that the sequence-processing layers ignored the padded portions of each cochleagram. The second layer was an LSTM layer with 512 hidden nodes that were fully recurrent. The final layer was a dense layer that converted an input vector X into an output vector Y of the length *n*, where *n* is the number of target classes (812, 1,007, or 1,819). With the output layer, we used the sigmoid activation function, which returns a value between 0 and 1 centered around 0.5. We used mean squared error loss (with a batch size of 100) to compute the mean of squares of errors between labels and predictions. For optimization during training, ADAM (Adaptive Moment Estimation) (Kingma and Ba, 2014) was used with a constant learning rate of 0.0001. Each word had ten tokens (the same words produced by ten different speakers), and nine of them were used for training and one for validation (nine-to-one train/validation split). Moreover, the networks were trained for 10,000 epochs (full passes over the training set).

### 4.4. Testing

All models were run ten times to ensure replicability. During the training, we checkpointed each of these ten iterations every 100 epochs to later reload the model and calculate accuracy metrics as training time increased. We then computed the cosine similarity (which ranges from 0 to 1) of the predicted target vector at the final time step of each word to the true target vector to quantify the distance between the predicted vector and the true vector of the target word in the lexicon. We selected cosine similarity rather than a simple binary cross-entropy threshold value because it is more conservative and psychologically more relevant (Magnuson et al., 2020). We reported the average cosine similarity (for all words) for every 100 epochs for both training and validation data. In addition, to test whether each model's training and validation accuracy was significantly different, we used the ANOVA function in R (R Core Team, 2023) to perform a two-way ANOVA between accuracy rates (cosine similarity), network type (3 levels: Dorsal, Fused, Ventral) and test type (2 levels: Training, Validation) with an interaction term.

To report word identification accuracy (the number of words the model correctly predicted), we also calculated the cosine similarity of the predicted vector at the final timestep of each word to all the other word vectors to quantify the distance between the predicted vector at the final time step relative to all other words in the lexicon. For every word in the training and validation set, the output layer of a model using the sigmoid activation function outputs a predicted vector. We took this output vector and compared it to the true vectors of all the possible words in the lexicon using cosine similarity. If the cosine similarity of the predicted vector and true vector is higher than the cosine similarity of all the comparisons, we deemed that the model correctly predicted the target word. In other words, we operationalized an accurate response as one in which the cosine similarity of the predicted target vector to the true vector was greater than that of all other words in the lexicon. For example, if the cosine similarity between the predicted word and the target word is 0.95, but it is not the highest cosine similarity (meaning that some other word vector is more similar to the predicted vector) we did not count it as a correctly predicted word. We aimed to show how accurate the model is on identifying the words when there are very similar candidates that compete with the target word. We reported this as the number of words that a model correctly predicted.

### 4.5. Generalization tasks details

Our aim was to compare the degree to which the featural representations of words discovered by the hidden layers of each model reflected hypothesized dorsal vs. ventral stream properties. We do not expect the fused network to be optimized for either articulatory or semantic representation. We chose one task for each featural representation. Articulatory properties, which we hypothesized would be captured more directly by the dorsal network, were examined using a classification based on the onset phoneme of each word. Onsets play a crucial role in identifying words through their articulatory features. Spoken word recognition relies heavily on word onsets (Marslen-Wilson and Tyler, 1980; Marslen-Wilson and Zwitserlood, 1989; Allopenna et al., 1998). We classified onsets based on the manner of articulation as vowels, voiced and voiceless stops, fricatives, nasals, liquids, and glides. We could have created more classes by splitting the vowels or fricatives into more classes (i.e., front, center back vowels or voiced, voiceless fricatives), but we preferred to have more balanced sets. Thus, we had seven categories in total.

Semantic properties, which we hypothesized would be captured more directly by the ventral network, were examined using a classification based on the part of speech category (POS) of each word. The POS category of a word, also known as its syntactic category, plays a crucial role in determining its meaning. POS category of a word is closely related to its conceptual category, or the category to which it belongs in the speaker's mental representation of the world (Lakoff, 1987; Jackendoff, 2002). We categorized words into singular and plural nouns, adjectives and comparative adjectives, base, past, gerund, and present verbs, and adverbs (nine categories in total). We chose to use nine categories rather than four general POS classes (noun, verb, adjective, adverb) because we wanted to test whether the networks learn morphological cues and differentiate, for example, singular nouns from plural nouns.

Analyses of generalization tasks were run based on the best performing iteration of the model runs. We decoded the words in our lexicon from the activations extracted from all three networks to check whether representations optimized for one task would support the other. Specifically, we extracted hidden layer activation patterns for 8,830 words (cochleagrams) categorized into 7 (articulatory task) and 9 (semantic task) classes, respectively. The features (hidden layer activations) were extracted from all three models at every time point (0 to 225) and then were standardized by removing the mean and scaling to unit variance. To quantify the decoding accuracy of activations of each network, we used Agglomerative Hierarchical Clustering with Euclidean distance and Ward (Ward, 1963) linkage methods [see Maimon and Rokach (2006) for a review of clustering methods] for each task. It is an unsupervised learning technique that groups similar data points using a bottom-up method, such that the points in the same group are more similar to each other than the points in the other groups. We used unsupervised learning to find patterns and relationships in the data without assuming a clear relationship between the input features (hidden unit activations) and any given output label. Decoding (clustering) performance was then evaluated using the adjusted Mutual Information score (AMI), a measure of the similarity between the true and predicted labels adjusted for chance (Vinh et al., 2010). The output ranges from [0,1], where one indicates perfect similarity between two label assignments, and random label assignments would produce a value of zero. AMI is also adjusted for a chance so that unbalanced class labels do not cause an issue. We used AMI over other clustering accuracy metrics (i.e., silhouette score, rand index, etc.) because it gave more robust results regardless of the number of clusters. These decoding steps were done in Python using the *numpy* and *sklearn* libraries (Pedregosa et al., 2011). Moreover, to test whether each model's decoding accuracy was significantly different, we used the ANOVA function in R to perform a two-way ANOVA between decoding accuracy rates (AMI), network type (3 levels: Dorsal, Fused, Ventral), and generalization task type (2 levels: Onset Phoneme Monitoring, Part of Speech Categorization) with an interaction term.

### 4.6. Error analyses

We also examined the kinds of errors the three systems make when asked to identify individual words and see whether they break down as we might expect. We expect to see phonological errors (e.g., saying *mouse* instead of *house*) from the dorsal network, which mimics reproduction conduction aphasia following damage to SMG due to the dorsal network being trained on the mapping between sound and articulation. In contrast, we expect semantic errors (e.g., saying *fork* instead of *spoon*) from the ventral network, which correlates to transcortical sensory aphasia following damage to the pMTG.

To do this for each of the three network predictions, we used the same definition of accuracy (based on cosine similarity to all the words in the lexicon) described above in Section 4.4; the network makes an error when its predicted vector has a higher cosine similarity to a non-target word. We then calculated the Levenshtein Distance (LD) (Levenshtein, 1965) (the minimum number of addition, substitution, and deletion operations needed to transform one string to the other) between the phonological transcriptions of the true word and the incorrect word (a word with highest cosine similarity to the output when it is not the target) as a metric for phonological similarity. LD is a ratio that returns a number between 0 (no similarity) and 1 (perfect similarity). For example, when the true word is *acted*, and the predicted word is *active*, the LD ratio between these two words would be 0.73, meaning that these words are phonologically similar. As for the metric of semantic similarity, we used 300-dimensional semantic vectors for each word from a SkipGram (Mikolov et al., 2013) model trained on a 1.9 billion token English corpus consisting of a blend of English Wikipedia and the English Open Subtitles database (https://opus.npl.eu). We then calculated the cosine similarity between the true word's SkipGram vector and the incorrect word's SkipGram vector as a metric for semantic similarity.

We predicted that phonological similarity (LD) between the true word and error word would be higher for the dorsal network but lower for the ventral. Similarly, the semantic similarity (cosine similarity) between the true word's SkipGram vector and the error word's SkipGram vector should be higher for the ventral network but not for the dorsal. We also predicted that the fused network would mix phonological and semantic errors equally.

### 4.7. Hidden unit selectivity analyses

We used two selectivity indices (SIs) to measure the degree to which hidden units of each network encode information related to phoneme and morpheme representation. As we noted above, the dorsal stream mapping between sound and articulation is dependent on identifying phonemes, and the ventral stream mapping between sound and meaning is dependent on identifying morphological units. Thus, we hypothesized that the dorsal network's mapping of speech input to words would create a representation of phonemes in hidden units, and the ventral network's mapping would create a representation of morphemes. Thus, the two SIs that we used tested these hypotheses about the information content of hidden units.

The Phonemic Selectivity Index (PSI), adapted from Mesgarani et al. (2014) and Magnuson et al. (2020), quantifies the hidden unit's response to a target phoneme relative to all the other phonemes. We used consonant-vowel (CV) and vowel-consonant (VC) diphones to extract each hidden unit's response to each of the 39 English phonemes over a 0–100 ms time window after phoneme onset. The Morpheme Selectivity Index (MSI) quantifies the selectivity of each hidden unit's response to a target morpheme relative to all the other morphemes. We used all the root-plus-one-affix words in our lexicon to extract each hidden unit's response to each of the 20 morphemes over the full-time window.

We extracted the hidden unit activations for each network (dorsal, fused, and ventral) from the best-performing model's epoch with respect to validation accuracy. Selectivity indices were then calculated for each hidden unit by counting the number of times that a target member of a class of phonemes or morphemes produced a response at least 0.3 activation units stronger than the nearest activation for a non-target class token. Values were standardized on a 0–1 scale based on the number of tokens bearing the target in each class. Once we obtained these selectivity matrices (of size item x hidden units), we used hierarchical clustering with Ward linkage and Euclidean distance to analyze them [see Magnuson et al. (2020) for a detailed overview of methodological choices].

We used PSI values to cluster phonemes based on phonetic features and tested whether the hierarchy of phonemes produced by each model follows the Sonority Hierarchy (Clements, 1990), where speech sounds are ranked based on their loudness (vowels > glides > liquids > nasals > voiced fricatives > voiceless fricatives = voiced stops > voiceless stops). We also compared the resulting phoneme hierarchies (dendrograms) from each network to the hierarchy of phonemes in English (Lee and Hon, 1989; Dekel et al., 2004; Pfeifer and Balik, 2011) and obtained correlation values using cophenetic correlation in the *dendextend* package (Galili, 2015) in R.

We used MSI values to cluster morphemes based on the POS category of the words that were created after the morphological transformation. For example, the plural morpheme' -s' is an inflectional morpheme attached to nouns and creates (plural) nouns, whereas the suffix “-ment” is a derivational morpheme and is attached to verbs to create nouns. Here, both morphemes create nouns, and we tested whether the hierarchy of morphemes produced by each model cluster morphemes that create the same part of speech category together.

### 4.8. Replicability, hardware, and software

Replicability was confirmed by repeating the complete training of all models (dorsal, ventral, and dual) ten times; only minor variations were observed between iterations. Simulations were conducted on a Linux workstation with an Intel(R) Xeon(R) Gold 5218 CPU running at 2.30 GHz, with 98-GB of RAM, and using an NVIDIA Quadro RTX 8000 (48-GB) graphics card. Simulations were conducted using Python 3.6, TensorFlow 2.2.0, and Keras 2.4.3. Each model required approximately 48 h (except the fused network, which took 96 h) to train on this workstation. The GitHub repository (https://github.com/enesavc/lstm-lex) provides an up-to-date container with all necessary explanations and jupyter notebooks for running our training code and analyses.

## 5. Results

### 5.1. LSTM classification accuracy

**Figure 3:**
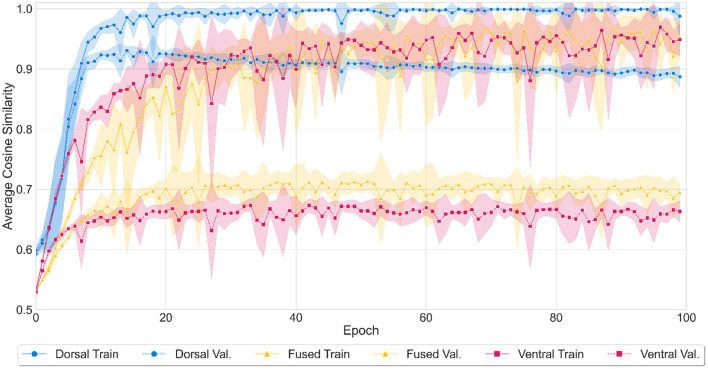
Model performance during the training of three (dorsal, fused, and ventral) models. Training performance over epochs was represented with solid (dorsal network with blue, fused network with yellow, and ventral network with red) lines and validation performance with dashed lines. Shaded areas represent standard deviation from mean accuracy for ten iterations of the model. The average cosine similarity between the predicted vectors and true vectors was computed for each model at every 100th epoch between 0 to 10,000 epochs.

All three models achieved high accuracy by the end of 10,000 training epochs (see Figure 3). The dorsal model's performance reached a mean average cosine similarity of 0.97 (SD = 0.08) for training and 0.89 (SD = 0.06) for validation. The model correctly identified 8,397 out of 8,830 words (95% accuracy) in both the training and validation sets combined, as assessed by the number of words where the cosine similarity of the predicted output to the target word was larger than the similarity to any other word in the lexicon. The ventral model's performance reached mean average cosine similarity of 0.90 (SD = 0.09) (training) and 0.66 (SD = 0.03) (validation), correctly recognizing 8,217 words (93% accuracy). The fused network reached a mean average cosine similarity of 0.89 (SD = 0.11) (training) and 0.69 (SD = 0.04) (validation), identifying 8,267 words (94% accuracy) correctly. It should be noted that the average cosine similarity shows the degree of similarity between the predicted vector and the true vector but is not directly equal to the word recognition accuracy since accuracy depends on the cosine similarity of the target word to all the words in the lexicon.

ANOVA results showed that there was a statistically significant difference in average accuracy by both network types (dorsal, fused, ventral) (*F*(2) = 2608.4, *p* < 0.001) and by test type (training, validation) (*F*(1) = 7956.4, *p* < 0.001). There was also an interaction between the two variables (*F*(2) = 639.8, *p* < 0.001). A Tukey *post-hoc* test revealed that the dorsal network showed higher accuracy on average than the fused network (14.5% more accurate) and a higher accuracy on average than the ventral network (15.3% more accurate). In addition, the fused network showed higher accuracy on average than the ventral network (0.08% more accurate). Training and validation accuracy differences were also significant, with training producing higher accuracy on average of 17.4% over validation. All pairwise comparisons were significant with *p* < 0.001.

### 5.2. LSTM generalization accuracy

The aim of the generalization tasks was to determine whether the dorsal and ventral networks or the fused network discovered different features and whether those features were independently optimized to support hypothesized dorsal vs. ventral stream processing. Specifically for the fused network, we hypothesized that being trained on both tasks simultaneously should be harder; therefore, the fused network should not master individual tasks as well as the other two models. We investigated whether the resulting feature spaces of each network trained on one task would support the other task by decoding the featural representation of each word based on the activation patterns in the LSTM layer of each network. For the onset phoneme monitoring task, activation patterns served as the input to a clustering analysis to identify seven manner-of-articulation classes (for testing dorsal function), or nine parts of speech categories for the POS categorization task (for testing ventral function).

**Figure 4:**
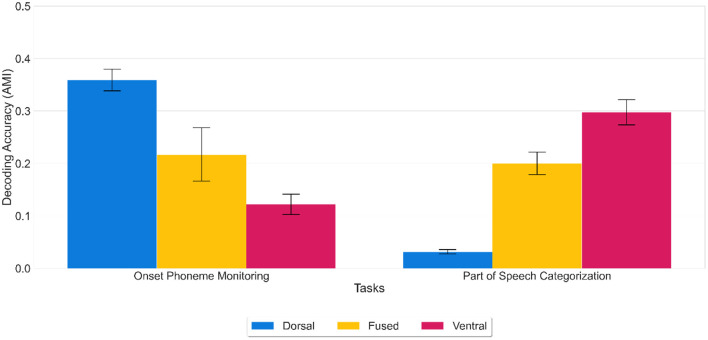
Decoding accuracy on generalization tasks. Decoding accuracy of dorsal, fused, and ventral networks on onset phoneme monitoring and POS categorization tasks using activation patterns extracted from the LSTM layer. Error bars indicate standard error of the mean. While the fused network (yellow) was moderately successful in each task, the dorsal network (blue) outperforms the ventral network (red) in onset phoneme monitoring and vice versa for POS categorization. Thus, the representations learned for one task do not buttress the other. Decoding accuracy was calculated using the AMI score (chance level 0%) for the overall time window (stacked all the temporal features up into one big vector in the shape of 1 X 226*512 from 0 to 225 (the offset of the cochleagram).

Results showed that onset phonemes could accurately be decoded from the dorsal network [mean decoding accuracy (AMI) of 0.36 (SD = 0.02)] and POS categories from the ventral network [mean AMI score of 0.30 (SD = 0.02)]. At the same time, the ventral network performed significantly worse [mean AMI score of 0.12 (SD = 0.02)] at onset phoneme discrimination than the dorsal network and vice versa for POS categorization (dorsal network, mean AMI score of 0.03 (SD = 0.01) (see Figure 4). Likewise, the fused network performed worse than the dorsal network [mean AMI score of 0.21 (SD = 0.06)] at onset phoneme discrimination and worse than the ventral network [mean AMI score of 0.20 (SD = 0.02)] at POS categorization.

ANOVA results showed that there was a statistically significant difference in average decoding accuracy by generalization task type (*F*(1) = 63.805, *p* < 0.001) and an interaction between the network type and generalization task type (*F*(2) = 433.771, *p* < 0.001). A Tukey *post-hoc* test revealed that generalization task type difference was significant (*p* < 0.001) with onset phoneme monitoring resulting in a higher decoding accuracy on average of 0.06 over part of speech categorization. The findings that networks trained on whole word articulatory information (sound to articulation) do not perform well on POS categorization, and networks trained on semantic information (sound to meaning) do not perform well on onset phoneme discrimination show that task-specific representations are required for generalization. Thus, the discovered features are not transferable. To perform well on the POS categorization task, a network should discover features that represent the POS category of a word in its hidden units. Similarly, to perform well on the onset phoneme discrimination task, a network should discover features that represent phonemes of a word in its hidden units. Our results on generalization tasks showed that the dorsal network discovered the category of onset phonemes, and the ventral network discovered POS categories even though they were not trained on this information directly. In other words, the dorsal features had an advantage for categorization related to articulation but not semantic categorization, whereas ventral features had an advantage for semantic categorization but not categorization related to articulation. As for the fused network, it performed equally on both tasks: it was worse than the dorsal network but better than the ventral network on onset phoneme discrimination and vice versa on the POS categorization task. This finding implies that the fused network could not discover task-specific features compared to the other two networks. To sum up, generalization tasks showed that the dorsal and ventral networks, but not the fused network discovered unique features from the same sound input; thus, representations developed for one task do not support the other.

### 5.3. The error patterns of networks

**Figure 5:**
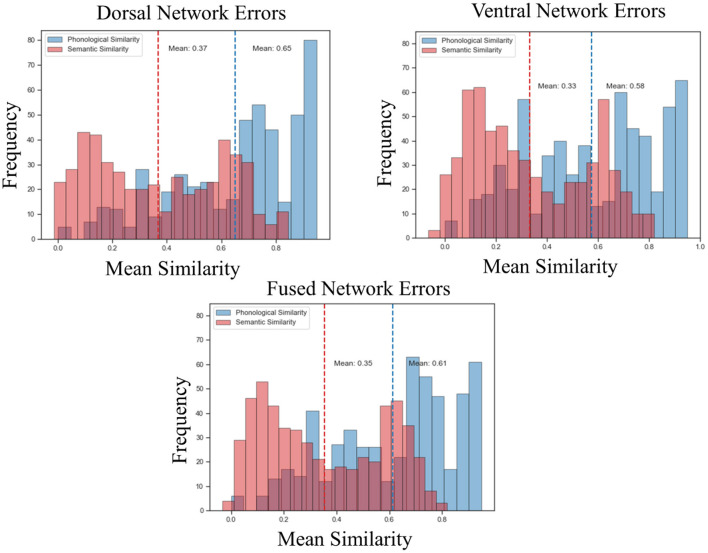
Error analysis. Phonological similarity *via* LD and semantic similarity *via* cosine similarity (from SkipGram) were calculated using the dorsal, fused and ventral errors. The x-axis of the plots shows the mean of the phonological and semantic similarity metric, and the y-axis shows the frequency distribution. The dotted vertical lines represent the means.

Both the dorsal and ventral networks discovered task-specific features and performed very well on their domain-specific generalization tasks. However, that was not the case for the fused network. The feature space discovered by the fused network did not support high task performance on generalization tasks. To better understand this performance difference, we analyzed the error patterns of each network. For each network, we calculated the phonological similarity (*via* normalized Levenshtein distance) between each of the incorrectly predicted words (the cosine similarity between the predicted vectors and true vectors was lower for target words than for at least one competitor word) and their true counterparts. The average phonological similarity between the dorsal network's errors was 0.65 on a scale of 0 to 1, meaning that the incorrectly predicted words tended to be phonologically similar to the target. For the same set of words, the average semantic similarity (cosine similarity between the vectors coming from SkipGram) was 0.37 (Figure 5). These results demonstrate that dorsal network errors were more on the phonological side of the continuum, confirming our hypothesis with regard to the dorsal network. The average phonological similarity for the ventral network between the incorrect words and their true counterparts was 0.58, and the average semantic similarity was 0.33. As for the fused network, the average phonological similarity was 0.61, and the average semantic similarity was 0.35 (Figure 5). These results show that the errors made by the ventral and fused networks were also more phonological and less semantic, in contradiction to our predictions based on the aphasia literature.

### 5.4. Hidden unit sensitivities

We have shown that each network could identify words equally well, and decoding analysis showed that the dorsal network discovered hypothesized articulatory features, whereas the ventral network discovered semantic features. However, surprisingly each network showed similar error patterns meaning that whenever a model makes an incorrect prediction, it is phonologically similar to the target word. We used selectivity indices (SIs) to examine the inner mechanisms of the networks.

While the dorsal network learned to map auditory speech input to vectors that represent phonological properties, the ventral network learned to map the same speech input to vectors that represent semantic properties. Our hypothesis was that once learning was successful, the hidden units of networks would discover some task-specific features. In particular, the dorsal network's mapping speech input to words might have created an implicit representation of phonemes in hidden units. Similarly, the ventral network might have developed representation for unique morphemes through morphological parsing of semantic interpretation of words. The fused network, on the other hand, would not be as task-specific as the other two networks because learning the phonemes and morphemes simultaneously would be hard. We tested these hypotheses using PSI and MSI measures of hidden node selectivity in each model combined with hierarchical clustering analyses of those measures. We then compared the resulting clusters to English phoneme and morpheme hierarchies to quantify the similarity.

**Figure 6:**
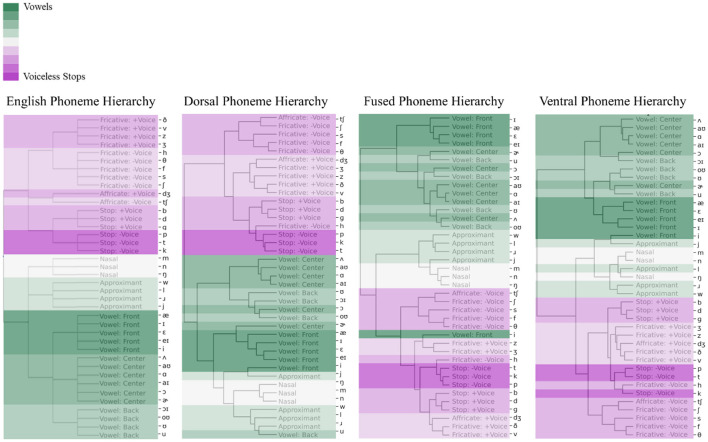
Phoneme hierarchies based on phoneme selectivity index (PSI). PSI values were used to cluster 39 English phonemes based on phonetic features. Phonetic features are color-coded following the sonority hierarchy: Dark green represents Front vowels and dark purple represents Voiceless-Stops. English phoneme hierarchy (left) were used as a baseline for the comparison of dorsal, fused, and ventral network's resulting phoneme hierarchies.

PSI results in Figure 6 showed that while all three networks developed some sense of phonetic information, the dorsal network hidden unit activations clustered phonemes better than the other two networks (dorsal phoneme hierarchy is more similar to English phoneme hierarchy). To quantify how faithfully a cluster hierarchy outputted by a model preserves the pairwise distances between the English phoneme hierarchy, we used cophenetic correlation. The results showed that the cophenetic correlation coefficient between the dorsal network's phoneme hierarchy and the English phoneme hierarchy was 0.72, implying that they were similar. In contrast, it was 0.56 for the fused model's hierarchy and 0.57 for the ventral model's hierarchy (the cophenetic correlation coefficient between the fused hierarchy and ventral hierarchy was 0.88).

The ventral and fused networks clustered approximants and nasals (sonorant sounds) together with obstruents rather than vowels. In addition, the fused network hidden unit activations wrongfully clustered the high front vowel (/i/) with voiced fricatives. The ventral network clustered the high front vowel (/i/) with the glide or semi vowel /j/ under a more general cluster of approximants and nasals. The dorsal and ventral networks perfectly clustered the obstruents into two big categories as the voiced and voiceless obstruents. This voiced vs. voiceless obstruent distinction was not perfect in the fused network. Nevertheless, each model showed a decent grouping of English phonemes following the Sonority Hierarchy. The success of the models in the grouping of phonemes might be related to the fact that they all received acoustic input in the form of cochleagrams (see Supplementary Figure S2 for the hidden unit activations from each network in response to 39 English phonemes).

**Figure 7:**
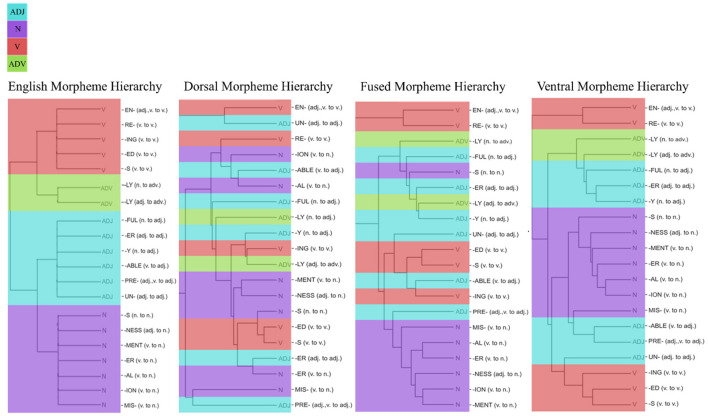
Hierarchies based on morpheme selectivity index (MSI). MSI values were used to cluster 20 English phonemes based on POS categories of the word. POS categories are color-coded: Light blue represents adjectives, purple for nouns, red for verbs, and green for adverbs. English morpheme hierarchy (left) were used as a baseline for the comparison of dorsal, fused, and ventral network's resulting morpheme hierarchy.

The MSI results in Figure 7 showed fundamental differences between the three models. MSI shows the selectivity of model hidden units to the 20 morphemes, which were coded based on the POS category of the word that was created after the morphological transformation. The clustering of morphemes based on dorsal hidden unit activations does not show a sensible grouping of morphemes, whereas the cluster from the fused network shows groupings of some nouns together. However, among the three networks, the ventral hidden units showed the best classification of the morphemes (based on the grammatical category of the words they create), where all the nouns are clustered together with adjectives (see Supplementary Figure S3 for the hidden unit activations from each network in response to 20 English morphemes). This shows that ventral stream mapping is dependent on identifying morphological units.

## 6. Discussion

Spoken language processing is organized broadly into parallel dorsal and ventral processing streams, and various lines of work show that each stream might have its own lexical interface area mediating mappings between acoustic-phonetic representation and stream-specific processing. The purpose of this paper was to determine why humans might have evolved two lexicons rather than a single lexicon that interacts with both processing streams. Specifically, we asked whether computational constraints on the mapping between acoustic-phonetic input and articulation vs. meaning create pressure for the development of different computationally efficient featural representations of words in the dorsal and ventral streams. Below, we discuss our results in the context of recent similar findings in the auditory systems that examine human speech recognition models and visual systems that examine functional specialization.

Our results demonstrated that training the networks on differently structured wordform representations produced different featural representations at the hidden layer of each model and that these emergent representations supported different patterns of performance on generalization tasks. While vectors that represent phonological properties were used as surrogates of articulatory representation in the dorsal network, and vectors based on patterns of lexical co-occurrence were used as surrogates of the ventral network, training on both patterns supported accurate identification of individual words (that each model showed successful training and validation performance). Both dorsal and ventral networks were successfully trained to map cochleagrams of tokens of spoken words onto output vectors representing words and to generalize that mapping from trained to untrained tokens. The generalization capacity of both models was also high, making the models predict more than half of the words in the validation set correctly. To this extent, both models learned to recognize a large set of spoken words produced by multiple talkers and map each token to the correct type successfully. The fused network's training and validation accuracy were comparable to the ventral network but worse than the dorsal network. Our interpretation of this finding is that training the network simultaneously on two different output vectors might have helped it to discover a feature set that allows word identification, but at the same time, this feature set is not as task-specific as the other two networks. Therefore, even though the fused network identified wordforms comparable to the other two networks, the representations it discovered were not as well optimized for specific mappings as the specialist dorsal and ventral models.

Despite being trained on output vectors that did not explicitly require phonemic representation, the dorsal network discovered a feature space that supported the classification of word-initial phonemes by articulatory class. Furthermore, hidden unit sensitivities of the dorsal network revealed that the model discovered the phonetic features of English phonemes clustered in a way that follows the sonority hierarchy. This result is consistent with the findings reported by Bhaya-Grossman and Chang (2022) examining human STG encoding distinct acoustic-phonetic and prosodic features. Similar findings were also reported by the EARSHOT model (Magnuson et al., 2020) in which hidden node selective sensitivity to both phonemes and articulatorily referenced phonetic features were shown to pattern with selectivity found in electrode recordings in human STG using similar input and model architecture. Although the hidden unit sensitivities of the fused and ventral networks also revealed the emergence of phonetic feature sets, neither of the model's features supported the successful classification of word-initial phonemes by articulatory classes as well as the dorsal network. We attribute the dorsal model's insensitivity to the POS categories to the failure to create a task-specific feature space that supported semantic mappings. This finding demonstrates the importance of task-optimal representation. Decent phonemic representations emerged spontaneously in each network trained on spoken words, but the dorsal network trained on phonological properties showed better generalization related to representations that are more rooted in articulatory properties.

In the same vein, the ventral network discovered a feature set that supported POS categories that play a critical role in determining a word's meaning despite having been trained on output representations that did not explicitly describe grammatical categories. Although the ventral network was trained on output vectors with structures that reflect the overlapping distributional patterns of individual words in meaningful contexts, the hidden unit selectivity of the model showed the emergence of grammatical classes of words. This finding is consistent with a broad body of work in distributional semantics and the findings of Elman (2004), whose recurrent network showed clustering in feature space that reflected grammatical category after training on a word prediction task. The finding that the ventral network outperformed the dorsal network on grammatical category classification is not unexpected, but it again demonstrates that task demands shape feature spaces that are better suited for different types of generalization. In addition, the ventral network showed better clustering than the dorsal network with respect to the POS category of words created by different derivational and inflectional morphemes. This shows that the ventral stream mapping between sound and meaning is more sensitive to morphological units.

We also closely investigated the kinds of errors models make when asked to identify individual words to see whether they pattern with dissociations in aphasia. Reproduction conduction aphasia, caused by damage to the inferior parietal lobe, is an acquired language disorder where phonological production shows phonological errors in tasks requiring spoken output (Franklin et al., 2002). This disorder is attributed to the degradation of dorsal representations. Similarly, semantic paraphasia seen in transcortical sensory aphasia following damage to posterior middle temporal regions (Fridriksson et al., 2009) is attributed to the degradation of ventral representations. We have found evidence for this hypothesis in the type of errors our dorsal network made. However, the error patterns of ventral and fused networks also exhibited more phonological errors than semantic errors. For example, all the models incorrectly predicted the target word *killing* as *killed* (even though, as experimenters, we expected an error like *murdering* for *killing* from the ventral network). While the phonological similarity between *killed* and *killing* is 0.62, the semantic similarity is 0.65. This example shows that since our lexicon includes lots of morphological derivatives of base lemmas (e.g., *kill, killer, kills, killing*, etc.), we are unlikely to find purely phonological or purely semantic errors. When the dorsal network makes a phonological error, most of the time, it is a semantic error as well; and when the ventral network makes a semantic error, in many cases, it chooses a phonologically similar word. In other words, if the model chooses the wrong form, then that error is phonologically and semantically related to the correct word in many cases, irrespective of what the model is optimized for during training. Thus, we think that the morphological complexity of the lexicon is what makes these error patterns look the way they do.

The fused network, although it performed very well during training and validation, did not do well on onset phoneme or POS categorization tasks. The goal of the onset phoneme categorization task was to test the degree of sensitivity of a network in response to the word-initial phoneme, which plays a key role in sound-to-form mapping. The onset of a word has a special status in lexical access of spoken words because it determines the level of activation between the competitors in the lexicon (Marslen-Wilson and Zwitserlood, 1989; Jusczyk and Luce, 2002). On the other hand, with the POS categorization task, we aimed to observe whether a network learns the grammatical category of a word which plays a key role in the mapping of sound to meaning [see Chiche and Yitagesu (2022) for an overview of the importance of POS tagging in NLP and its impact on meaning extraction]. The fused network's performance on these two tasks was worse than the other two networks. Is this due to the lack of task-optimization or the specific nature of these two tasks? We propose the possibility that the fused network could not find a common feature space to solve both tasks (cf. Dobs et al., 2022). Although it may have formed representations that are partially phonemic and partially semantic, the feature space it built over the training process does not have the task-specific information to eclipse the performance of the other two networks. This behavior can be explained as an adaptation to increased processing costs where the fused network is adapting its weights to forget some of the phonemic representations in order to keep and build semantic representations (or vice versa) concurrently. Of course, future work that tests such a network on many different tasks will ground these preliminary results.

Overall, the results on the generalization tasks and the hidden unit SIs show that the dorsal features were more successful on an articulatory task and the ventral features were more successful on a semantic task indicating that representations optimized for one task would not be transferable to the other. One critical design feature of this study is that each network gets the same sound input but is paired with different output vectors. Therefore, each network is supposed to develop different features in its hidden layer representation. The results showed that the dorsal network discovered phonemes, and the ventral network discovered morphemes without explicit training. And both networks were successful on related tasks due to these task-specific representations. If task-specific representations were not discovered, both the dorsal and ventral networks would have resulted in comparable performance on generalization tasks. For example, the fused network, which was trained on both articulatory and semantic information simultaneously, could not discover task-specific features and showed comparable performance on generalization tasks.

These results suggest that lexical interface areas in the dorsal and ventral pathways of the brain may have arisen from computational constraints for optimizing the primary mapping functions that support lexically organized processes in the dorsal and ventral processing streams. Our results align with previous research in the visual domain, demonstrating how computational constraints can give rise to functional specialization. Dobs et al. (2022) used deep CNN models to ask why are face and object processing segregated in the human brain and test whether functional specialization is a good design strategy in the first place. They hypothesized that the functional specialization might be due to three possibilities: (i) it resulted by accident of evolution, (ii) it is the output of time selective modulation, and (iii) computational reasons. They tested this third hypothesis and trained two separate networks with the same architecture on the categorization of faces and objects. The results revealed that the network trained on faces performed worse on object categorization than the object-trained network and vice versa. In sum, Dobs et al.'s results, together with our results, support the claim that the optimization of features for a specific task might be a design feature of the human brain [see also Yang et al. (2019) for similar results on 20 different cognitive tasks using recurrent neural networks].

## 7. Conclusion

This study aimed to characterize the functional specialization of word representation and examine whether computational constraints inherent in the mapping between sound and higher-order linguistic representations (articulation vs. meaning) could have shaped the development of parallel lexical interface areas that rely on different featural representations. We showed that task-specific features discovered by LSTMs trained on vectors representing phonological properties supported articulatory classification better than those trained on distributional semantics. Conversely, featural representations of words from LSTMs trained on distributional semantics supported semantic generalization better than those trained on vectors representing phonological properties. These results support the claim that different featural projections of wordform may be needed to support efficient processing in the dorsal and ventral speech streams. Thus, we showed that the characteristics of the input data determine the representations that the machine/algorithm (and potentially the brain) must uncover for feature detection. In future work, we hope to ground these analyses via direct comparisons between patterns of human cortical and machine classifier hidden node responses to spoken words.

## 8. Limitations of the work

This work was intended as a broad computational exploration of computational factors shaping wordform representation and not an explicit processing or neural model of human lexical processing. Human lexical processing appears to depend on interactive excitatory and inhibitory processes that we have not attempted to implement. While our lexicon is similar in size to those implemented word recognition models such as TRACE (McClelland and Elman, 1986) or EARSHOT (Magnuson et al., 2020), it is significantly smaller than the typical 20–100,000-word lexicon of an adult native English speaker. A more realistically scaled lexicon would pose much denser perceptual and semantic spaces, which might necessitate reliance on richer feature systems. In contrast to previous work, we have used complex morphology so that our lexicon reflects the English lexicon more naturally; we believe this is a strength of our study. However, our error analysis showed that including lemmas along with many of their derivatives, worked against our predictions by inducing a correlation between phonological and semantic similarity in the lexicon. Using a larger, carefully controlled lexicon (controlling phonotactic frequency, cohort size, phonetic saliency, phonological neighborhood density, length of the word, and semantic similarity of words) with natural speech might allow us to decouple phonological and semantic errors. In addition to lexicon design, we made a number of other choices to simplify implementation, including the restriction of words that play multiple grammatical roles (e.g., *act* can be a noun or a verb) to a single form in ventral coding, the use of synthetic speech that does not show naturalistic patterns of phonological reduction, and the use of minimalist representations of articulatory or morphosyntactic target vectors.

These choices allowed us to make an initial exploration of computational forces that make wordform representation tractable, but they preclude strong inferences about the neural representation of specific wordforms. Fine-grained validation of these results against neural data using methods such as representational similarity analysis will be an essential step in understanding the principles explored here. However, meaningful comparisons with human data will depend on the development of more realistic training sets and target representations. Our error analysis was a modest qualitative step in that direction.

While the work aimed to explore computationally optimal feature space, we recognize our models themselves may not have been optimal in several respects. By testing and modifying model parameters independently, our methods have failed to identify optimal sets of interacting parameters. Furthermore, we used a small subset of possible model architectures with loss, optimization, learning rate, number of layers, and hidden nodes. Thus, it is a possibility that a network with a different optimization strategy (i.e., Bayesian optimization or grid search) might perform well and show shared feature space for the mappings between sound to articulation and sound to meaning.

Our study cannot prove why the human brain discovers unique dorsal and ventral features for better speech perception. Instead, we can only argue that given the tested circumstances, we found functional specialization, which might reflect the functional specialization of the human brain hypothetically. Last, our results do not show anything about how the computational constraints interacted with potential anatomical and evolutionary constraints. Instead, we only showed the possibility that the computational constraints in the input data might have shaped the development of parallel wordform networks if they exist, as hypothesized by Gow (2012) and previous work. We hope that the arguments and hypotheses developed here will enable future cognitive scientists to ask more fine-grained questions about the language in the brain, considering the computational optimization as a design feature [see Kanwisher et al. (2023a) for an argument of using ANNs to ask “why” questions about the brain].

## Data availability statement

The datasets presented in this study can be found in online repositories. The names of the repository/repositories and accession number(s) can be found below: https://github.com/enesavc/lstm-lex.

## Author contributions

EA and DG conceived of the initial project and wrote the original draft of the manuscript. EA implemented the model and analysis scripts. EA, KB, and MH reviewed the software and data analysis. MH conducted the COCA corpus analyses. All authors contributed to the interpretation of results, development of the data analysis strategies, and to the writing of the manuscript. All authors contributed to the article and approved the submitted version.
