# Supplementary material for: A tale of two lexica: Investigating computational pressures on word representation with neural networks
Source: Front Artif Intell. 2023 Mar 27;6:1062230. doi: 10.3389/frai.2023.1062230 (PMC10083378; doi:10.3389/frai.2023.1062230)
Supplement: Supplementary file 1 [file Data_Sheet_1.docx]

Supplementary Material

# Model Optimization Details

We optimized model parameters by systematically varying one parameter at a time and comparing validation accuracy for the resultant models. For all model runs, our batch size contained 100 examples and we ran an extensive hyperparameter search over 1000 epochs for *loss function* [binary cross entropy (bce), mean squared error (mse)], *optimization algorithm* [stochastic gradient descent (sgd), root mean squared propagation (rmsProp) , adaptive moment estimation (adam)], *initial learning rate* values of [0. 01, 0.001, 0.0001, 0.00001], *number of recurrent layers* [1,2,3,4] and *number of hidden nodes* in each recurrent layer [32, 64, 128, 256, 512, 1024, 2048, 4096] (see Table 1 for details). For each parameter category, while we searched over the best value, we kept the other parameter values constant. For example, while we searched over the best loss function (bce vs. mse), we used *adam* as the optimization method with learning rate of 0.0001 with one recurrent layer with 512 hidden nodes.

**Table S1**: Details of hyperparameter testing. Optimal parameters for the validation set were bolded and used in the neural networks reported in the manuscript.

| **Parameters** | **Description** | **Values Tested** |
| --- | --- | --- |
|  |  |  |
| loss | compute the quantity that a model should seek to minimize during training | binary cross entropy, **mean squared error** |
| optimization | methods used to change the attributes of the model | stochastic gradient descent, root mean squared propagation, **adaptive moment estimation** |
| lr | Initial learning rate | 0.1, 0.001, **0.0001**, 0.00001 |
| nlayers | number of recurrent layers | **1**,2,3,4 |
| nhid | number of nodes in each recurrent layer | 32, 64, 128, 256, **512**, 1024, 2048, 4096 |

In the manuscript, we presented results for the best hyperparameter setting of each category. See Fig. S1 below for details on how model performance is influenced by different parameter values.


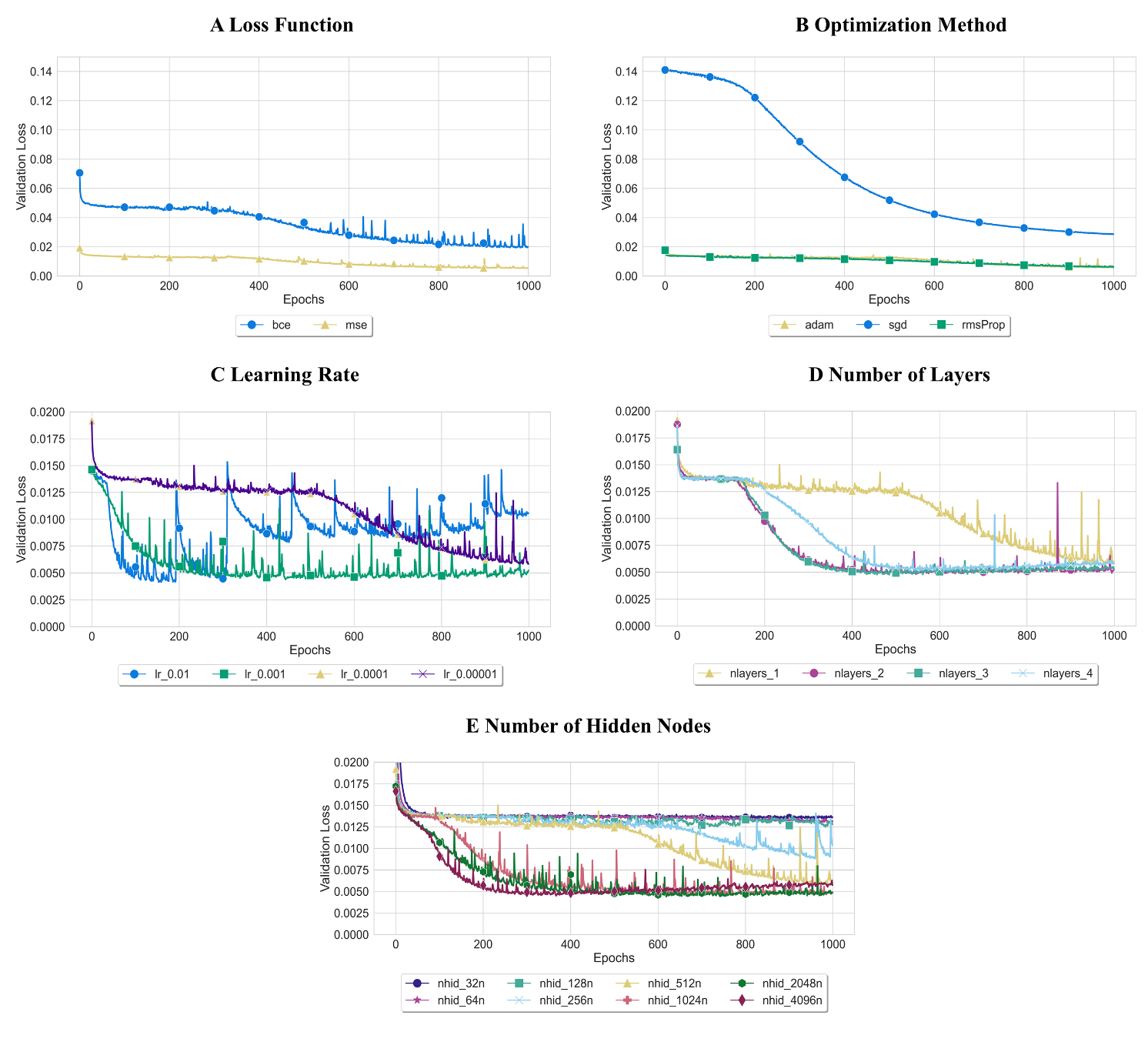


**Figure S1.** **Model Optimization Details. (A)** The choice of loss function reflected on validation loss. Mean squared error (mse) loss function yielded better validation loss than binary cross entropy (bce). **(B)** The choice of optimization method reflected on validation loss. Adaptive moment estimation (adam) optimization method yielded better validation loss than stochastic gradient descent (sgd) and root mean squared propagation (rmsProp). **(C)** The choice of constant learning rate reflected on validation loss. 0. 0001 as the constant learning rate yielded better validation loss than 0.01, 0.001 and 0.00001. **(D)** The choice of number of recurrent layers reflected on validation loss. While it is the case that it may be possible to train the larger models faster, they don’t do any better overall than the 1-layer model (which has the advantage of also being less complex). **(E)** The choice of number of hidden nodes reflected on validation loss. 512 fully recurrent hidden nodes yielded better validation loss than 32, 64, 128 and 256 hidden nodes. While the deeper models (1024, 2048, 4096) seem to result a lower validation loss faster, they don’t perform any better than the shallower model.

# Hidden Unit Selectivity Additional Result Heatmaps

The activations of the hidden units in each network (dorsal, fused, and ventral) were retrieved and selectivity indices were computed for each hidden unit by determining the number of times that a specific class of phonemes or morphemes produced a response that was at least 0.3 units stronger than the next strongest response from a non-target class token. These values were standardized on a scale of 0-1, considering the number of tokens that belonged to the target class. After obtaining these selectivity matrices (matrices of size item x hidden units), the matrices were analyzed using a hierarchical clustering method with Ward linkage and Euclidean distance (see Magnuson et al. (2020) for more information). Fig. S2 below shows the hidden unit activations from each network in response to 39 English phonemes, and Fig. S3 in response to 20 English morphemes.

| 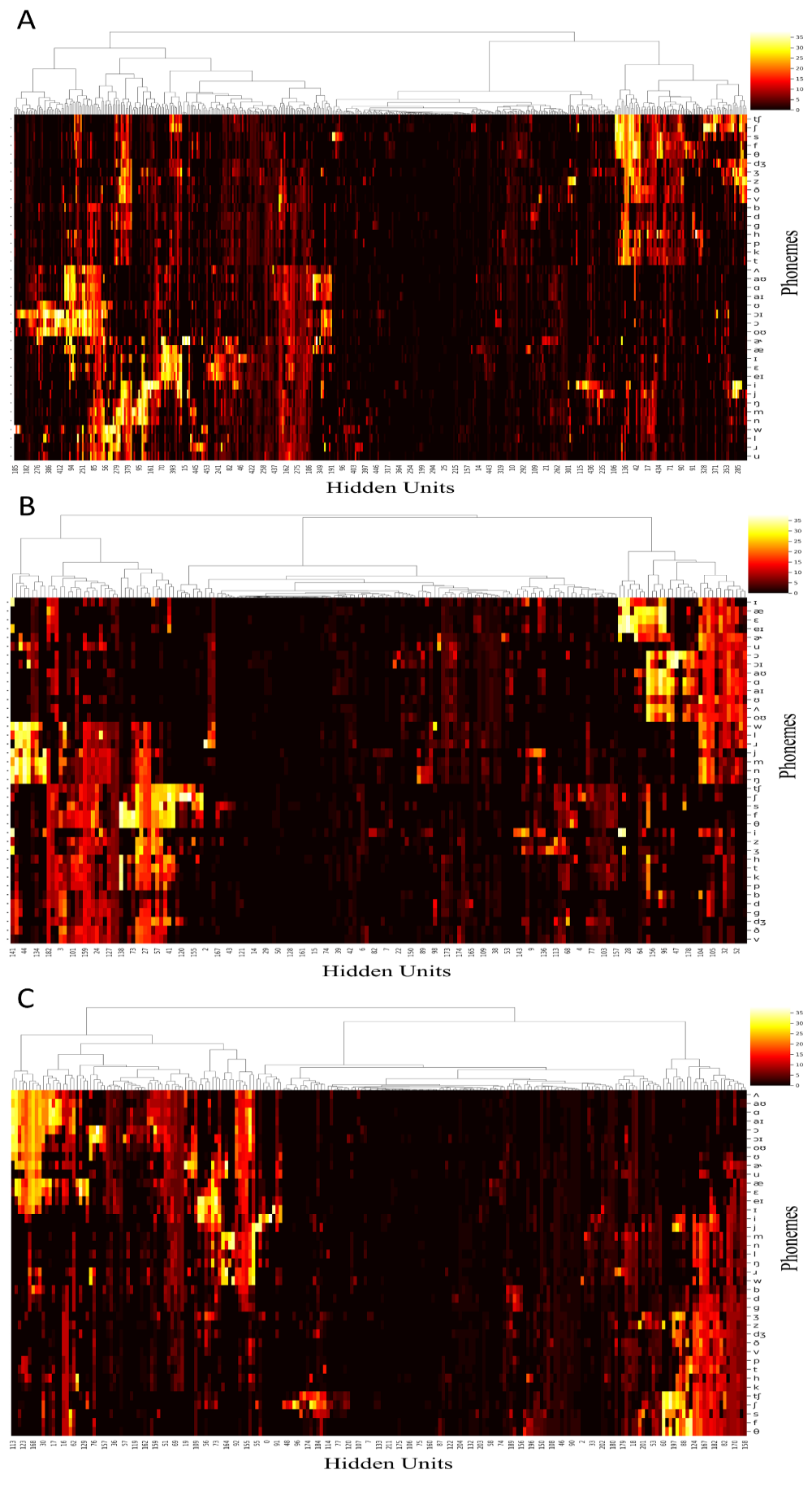 | **Figure S2. Phoneme Selectivity Index (PSI) Heatmaps.** Dorsal (A), Fused (B), and Ventral (C) models hidden unit representation of 39 English phonemes. The x-axis represents hidden unit responses, and the y-axis represents phonemes. If a hidden unit's response to a target phoneme exceeds its response to a non-target phoneme by a threshold of 0.3, PSI for that target phoneme receives 1 point. Yellow indicates high selectivity with a maximum PSI of 38. Out of 512 hidden units of the dorsal network, 454 with strongly selective responses are included (the rest of the hidden units were not sensitive) (183 from fused network and 217 from the ventral network). | |
| --- | --- | --- |
| **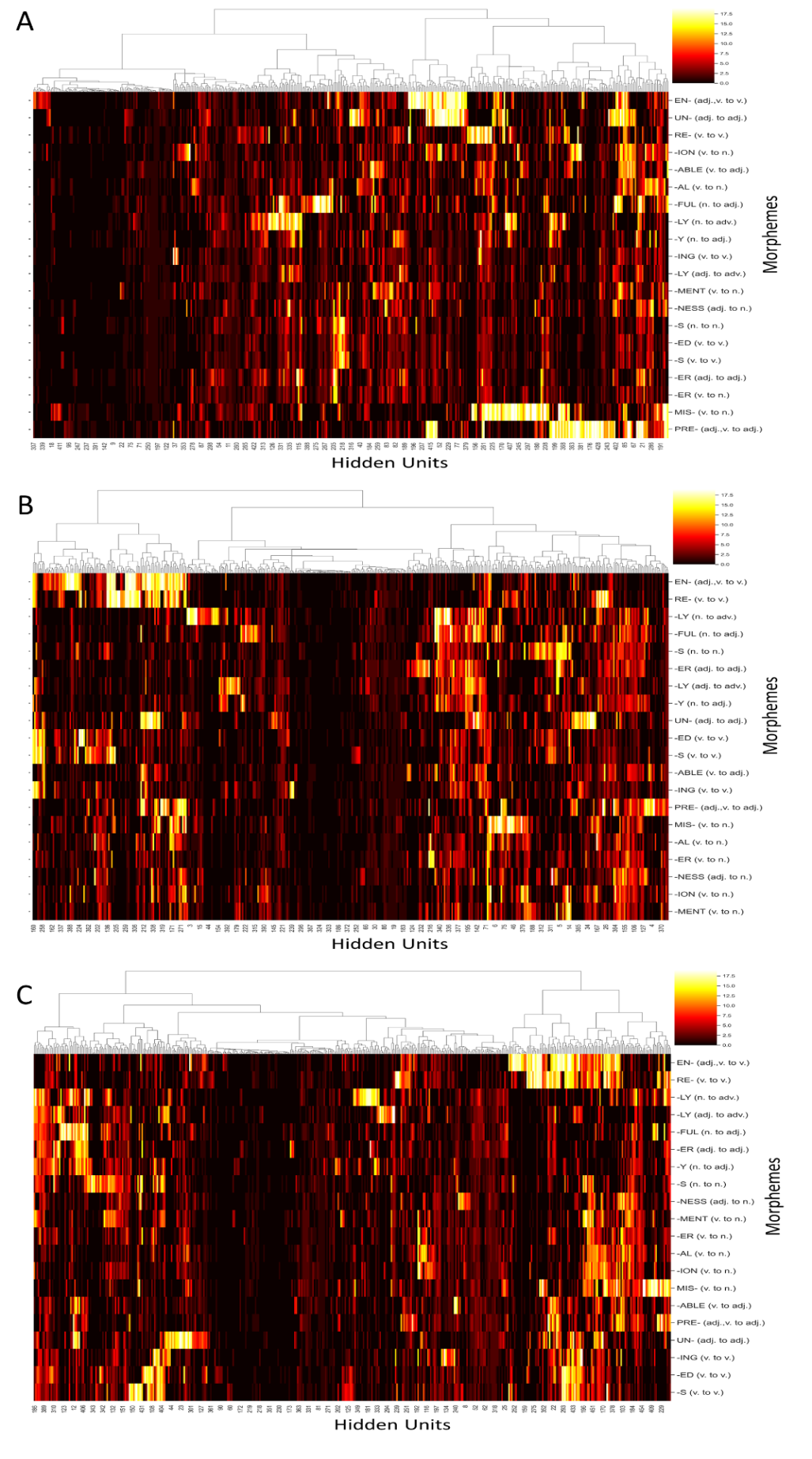** | | **Figure S3. Morpheme Selectivity Index (MSI) Heatmaps.** Dorsal (A), Fused (B), and Ventral (C) models hidden unit representation of 20 morphemes. The x-axis represents hidden unit responses, and the y-axis represents morphemes. If a hidden unit's response to a target morpheme exceeds its response to a non-target morpheme by a threshold of 0.3, MSI for that target morpheme receives 1 point. Yellow indicates high selectivity with a maximum MSI of 19. Out of 512 hidden units of the dorsal network, 432 with strongly selective responses are included (the rest of the hidden units were not sensitive) (413 from fused network and 455 from the ventral network). |
